# Obesity, COVID-19 and innate immunometabolism

**DOI:** 10.1017/S0007114520003529

**Published:** 2020-09-07

**Authors:** Laura E. Gleeson, Helen M. Roche, Frederick J. Sheedy

**Affiliations:** 1 School of Medicine, Trinity College, Dublin, Republic of Ireland; 2 Department of Respiratory Medicine, St James’s Hospital, Dublin, Republic of Ireland; 3 School of Public Health, Physiotherapy and Sports Science, University College Dublin, Republic of Ireland; 4 Institute for Global Food Security, Queen’s University Belfast, Belfast BT9 5DL, Northern Ireland; 5 School of Biochemistry and Immunology, Trinity College, Dublin, Republic of Ireland

**Keywords:** IL-1*β* inflammation, COVID-19, Immunometabolism, Obesity

## Abstract

As COVID-19 continues to spread worldwide, severe disease and mortality have been observed in obese patients. We discuss how obesity and obesity-associated factors such as ‘meta-flammation’, dietary fat intake and paradoxical suppression of the innate immune response within the pulmonary compartment may be crucial determinants in the host response to a novel viral pathogen. Modulation of immune cell bioenergetics and metabolic potential plays a central role in the innate immune response to infection, and as we strive to combat this new global health threat, immunometabolism of the innate immune system warrants attention.

## Graphical abstract

**Figure:**
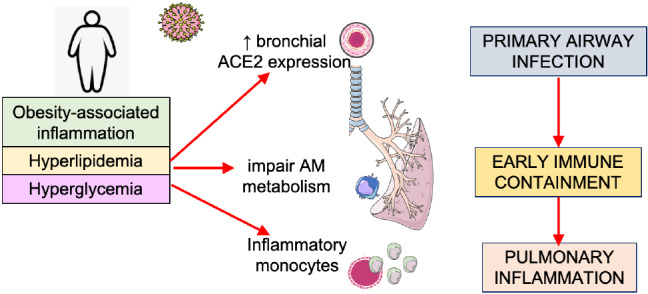

## The COVID-19 epidemic

As epidemiological data on the dramatic COVID-19 pandemic are disseminated, obesity has emerged as a major risk factor for severe disease necessitating intensive care unit (ICU) admission^(1–5)^. Obese patients <60 years old have a 2·2-fold increased rate of hospitalisation with coronavirus disease 2019 (COVID-19)^(2)^, while a 7·36 increased risk of mechanical ventilation has been reported in patients with BMI 35 kg/m^2^ or greater^(4)^. The observation of worse outcomes in obese patients was also seen during the 2009 H1N1 influenza pandemic, with 61 % of fatalities occurring in obese adults^(6)^. The apparent increased severity of viral pneumonia in this population is critically important given the increasing prevalence of obesity globally^(7)^. Multiple potential explanations for this association have been proposed, including altered pulmonary physiology in the obese patient, predisposition to thromboembolic complications which are observed with increased frequency in COVID-19 patients and increased incidence of co-morbidities that may impact upon outcome and render ICU management more complex and challenging^(5,8,9)^. However, a growing body of evidence implicates direct obesity-induced immune dysregulation. Increased lethality of H1N1 influenza observed in obese mice has been linked to impaired T-cell responses^(10)^, while failed influenza vaccine response is correlated with impaired antibody generation^(11)^. Likewise, human studies have demonstrated failure of influenza vaccine response in obese adults^(12)^. Similarly, increased susceptibility of obese patients to severe COVID-19 infection is hypothesised to be linked to impaired successful immune responses against the virus^(13)^, particularly through diet-derived reconfigurations of protective immunometabolic responses.

## Obesity and immune dysfunction

While obesity has been linked to dysregulated adaptive immunity and failure of antibody generation following infection or vaccination, severe acute respiratory syndrome coronavirus-2 (SARS-CoV-2) is a novel virus to which no one has an established adaptive immune response upon first exposure. Initial host defence against a novel virus relies entirely upon the rapid, non-specific innate immune response^(14)^. Thus, the impact of obesity upon innate immunity likely plays a crucial role in the severity of COVID-19 disease. Obesity creates a state of chronic inflammation, termed ‘meta-flammation’, characterised by sustained pro-inflammatory innate immune responses mediated through activation of the NLR family pyrin domain-containing 3 (NLRP3) inflammasome/IL-1 axis^(15–19)^. Adipose tissue directly secretes pro-inflammatory TNF*α*, IL-1 and IL-6, with downstream activation of the transcription factor NF-*κ*B, driving a state of oxidative stress^(20)^. This meta-flammation underpins the pathogenesis of several obesity-related conditions, including type 2 diabetes mellitus, CVD and non-alcoholic fatty liver disease^(21,22)^. Similarly, the establishment of ‘trained immunity’, a non-specific priming of progenitor cells of the innate immune system which alters their functional responses, is influenced by meta-flammation associated with these conditions^(23,24)^.

Despite increased basal levels of pro-inflammatory mediators, obesity-associated meta-flammation is paradoxically associated with attenuated innate immune response to both bacterial and viral infections^(25,26)^. Diet-induced obese mice demonstrate a substantially higher mortality rate than lean mice following influenza infection, with reduced expression of pro-inflammatory cytokines TNF*α*, IL-6, IL-1*β* and interferon-1 in the pulmonary compartment early post-infection^(27,28)^. Interestingly, however, by day 6 post-infection, the lungs of obese mice contained higher expression of TNF*α* and IL-1*β*, with similar expression of anti-inflammatory IL-10 to that of lean mice, suggesting a ‘skewing’ towards a pro-inflammatory state that results in lung tissue damage^(27)^. One possible explanation for this observation is failure of early macrophage responses in the obese mouse resulting in insufficient viral control by the innate immune system, with later T-cell infiltration causing increased inflammation and tissue damage that contributes to pathogenesis. Lipopolysaccharide-induced obese mice demonstrated impaired macrophage production of TNF*α*, IL-1*β* and IL-6 when challenged, with more severe lung pathology following infection with H1N1 influenza^(29)^. This mirrors the pathogenesis observed in severe COVID-19 disease, where early localised host responses fail to control viral replication, with later surges in inflammation – the ‘cytokine storm’, particularly driven by IL-6, which is associated with increased mortality^(30)^ – causing overt tissue damage that ultimately leads to respiratory failure. Elevated leptin levels characteristic of obesity may also contribute, with a murine model of 2009 H1N1 influenza infection demonstrating reduced IL-1 and IL-6 levels following administration of anti-leptin antibody to obese mice, resulting in improved survival^(10)^. Interestingly, though attenuated levels of pro-inflammatory cytokines were evident in the pulmonary compartment early post-infection in obese mice, systemic levels of TNF*α*, IL-1 and IL-10 were elevated at early time points similar to that of lean mice^(28)^.

## Macrophage metabolism and bioenergetics

We have previously described a similar state of baseline hyper-inflammation but paradoxical failure to mount an adequate innate immune response to infection in smokers’ pulmonary macrophages compared with those of non-smokers’^(31)^. Upon stimulation, healthy human pulmonary macrophages undergo a dramatic shift in central carbon metabolism, termed glycolytic reprogramming, with increased reliance on glycolytic metabolism rather than oxidative phosphorylation to generate energy^(32)^, resulting in activation of the NLRP3/IL-1 axis through consequent impact on transcription factor expression, diversion of metabolic intermediates into biosynthetic pathways and perturbations of the electron transport chain^(33,34)^. Similarly, ‘trained immunity’, the protective effect of which is being examined in the context of COVID-19 due to *Bacillus* Calmette–Guérin vaccination^(35)^, requires such glycolytic reprogramming in progenitor innate immune cells^(23)^.

Smokers’ macrophages, in contrast to non-smokers’, are skewed towards increased glycolytic activity at baseline^(36,37)^, with elevated IL-1 production, similar to the chronic hyper-inflammation observed in innate immune cells from obese patients. Despite this, however, smokers’ macrophages are unable to ramp up glycolytic activity and consequent pro-inflammatory and anti-microbial functions in response to pathogenic challenge, due to a state of ‘bioenergetic exhaustion’ induced by this chronic baseline activity^(37)^. Others have also demonstrated increased ‘polarisation’ of macrophages from smokers lungs at baseline^(38)^, notably describing increased proportions of M1-skewed macrophages, known to characteristically favour glycolytic metabolism. Interestingly, analyses of bronchoalveolar lavage fluid from SARS-CoV-2-infected patients have similarly identified higher proportions of recruited M1-like macrophages and depletion of lung-native tissue resident alveolar macrophages (which preferentially use oxidative phosphorylation to generate energy) in those with more severe disease^(39)^. Though baseline BMI was not reported in this study, strong epidemiological evidence suggests severe disease to be more prevalent in obese patients^(1,2)^ and existing studies have shown that obesity-associated metabolic signals alter tissue resident macrophage phenotype away from the classical anti-microbial programme^(40)^.

## The impact of obesity on innate immune responses

**Fig. 1.**
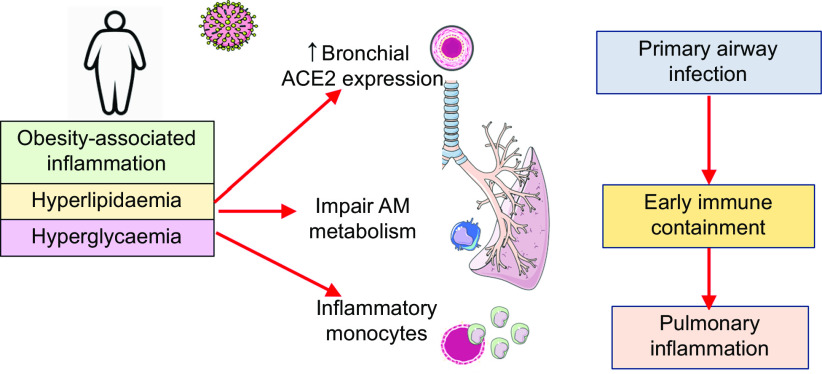
The trifecta of immunometabolic defects in the innate immune response to coronavirus disease 2019 (COVID-19) in obese people. Increased expression of angiotensin-converting enzyme-2 (ACE2), the receptor facilitating severe acute respiratory syndrome coronavirus-2 (SARS-CoV-2) infection, has been reported in the respiratory tract and lung of obese mice. Alveolar macrophage (AM) metabolism is altered by the lung environment and can be exhausted by baseline chronic inflammation affecting early microbial containment. Circulating bone marrow-derived monocytes can be affected by coincidental adverse obesity-related metabolic stressors, for example, poor blood glucose control, hyperlipidaemia. Resultant immune training in the bone marrow monocyte lineage leads to a hyperinflammatory phenotype that could facilitate up-regulation of disease-causing inflammation in the lung. This figure was created using Servier Medical ART templates, which are licensed under a Creative Commons Attribution 3.0 Unported License; https://smart.servier.com

Although many features of obesity can exhaust immune function through hyper-stimulation, ranging from hyperglycaemia, insulin resistance to hyperlipidaemia, we must consider the differential effect these factors have at various levels of innate immune function. Exhaustion of anti-microbial responses in resident lung macrophages could be coupled with hyper-stimulation or training of the immune system at the level of the bone marrow and circulating monocytes, which traffic to the lung after infection and trigger excessive inflammatory responses. Blood glucose control has been implicated in COVID-19 severity, with hyperglycaemic patients experiencing worse disease^(41)^. Thus, the effect of this increased glucose on immunometabolic functions is being examined. Ongoing and unpublished work examining the importance of glycolysis in circulating monocytes after SARS-CoV-2 infection indicates that excessive glycolysis may actually facilitate viral replication and drive excessive disease inflammation once recruited to the lung^(42)^. At the same time, recent murine studies of resident lung macrophages, however, suggest these cells preferentially use fatty acid oxidation rather than glycolysis to adapt to the lipid-rich and glucose-deprived environment in the lung^(43,44)^. Strategies which boost glycolysis and pro-inflammatory function specifically in these cells could be used to improve early anti-viral containment. Additionally, obesity has also been shown to alter the expression of the receptor for SARS-CoV-2 entry on respiratory epithelia, with retrospective analysis of angiotensin-converting enzyme-2 (ACE2) mRNA demonstrating increased expression in lung epithelia in obese high-fat diet-fed mice^(45)^. Thus, obese patients could suffer from a trifecta of immunometabolic defects at level of lung epithelium (primary infected cell), the alveolar macrophage (the primary responding cell) and circulating monocytes (the primary recruited cell), which increase their susceptibility to disease (Fig. 1).

## Obesity-associated inflammation and dietary quality fat intake

Obese phenotypes vary greatly, which may be partly attributable to diet quality and/or physical activity^(46)^. There is clear evidence that NLRP3 inflammasome-mediated IL-1*β* inflammation influenced by obesity has been outlined above. However, it is possible that this paradigm is more complex and linked to the quality/composition of the obesogenic diet. For example, the nature of different fatty acids and/or their metabolites may play a role. Excessive dietary fat presented as SFA greatly accentuates obesity-induced metabolic inflammation^(47,48)^. In contrast, feeding excessive MUFA or PUFA do not accentuate NLRP3 activity or IL-1*β* inflammation to same extent, possibly via re-configuration of key metabolic hubs including AMP-activated protein kinase^(48,49)^. We posit that certain dietary elements, such as SFA, fructose-induced endogenous SFA and/or their metabolites including ceramides may be particularly deleterious within the context of obesity-related immunometabolic dysfunction. And whilst hypothetical, this may in turn determine not only the nature of the innate immune response in COVID-19 but also the efficacy of vaccination at the population level^(11,12)^.

There are significant gaps in knowledge with respect to the impact of different dietary elements on COVID-19-related immunity (as recently reviewed^(50)^) and within the context of obesity. To this end, we need to be cautious and proceed with sound nutritional evidence. One case in point being the recent controversy wherein some observational studies linked low vitamin D status, which often coexists with obesity, to adverse COVID-19 outcomes. It is probable that vitamin D, alongside other important nutrients, plays an important role in immune cell metabolism and related immune functionality, as most cells can convert the vitamin D precursor into its active form. This does not imply that vitamin D supplementation in high-risk obese subjects can reduce risk. There are too many confounders underlying associations, not least of which vitamin D status is simply a good biomarker for several micronutrient deficiencies, contributing to malnutrition despite obesity.

## Future perspectives

The innate immune response to SARS-CoV-2 infection is likely crucial in determining the clinical course of COVID-19 disease, and obesity-associated dysregulation of this response is critically important given the high prevalence of this co-morbidity worldwide. While understanding the direct impact of obesity upon COVID-19 pathogenesis is important, from the diet and lifestyle perspective, there may be additional elements we could better understand which impact innate immune function at multiple levels. Based on parallels that can be drawn with our findings in smokers’ pulmonary macrophages, metabolic characteristics of innate immune response in the obese patient potentially represent a key feature of the unique susceptibility of this population to severe disease. As we struggle to develop therapies and vaccines to combat this new global health threat, immunometabolism of the innate immune system warrants attention from the global scientific community.
